# Exacerbation of Allergic Airway Inflammation in Mice Lacking ECTO-5′-Nucleotidase (CD73)

**DOI:** 10.3389/fphar.2020.589343

**Published:** 2020-11-30

**Authors:** Elisabetta Caiazzo, Ida Cerqua, Maria Antonietta Riemma, Roberta Turiello, Armando Ialenti, Jurgen Schrader, Giuseppe Fiume, Carmen Caiazza, Fiorentina Roviezzo, Silvana Morello, Carla Cicala

**Affiliations:** ^1^ Department of Pharmacy, School of Medicine and Surgery, University of Naples Federico II, Naples, Italy; ^2^ Department of Pharmacy, University of Salerno, Salerno, Italy; ^3^ PhD Program in Drug Discovery and Development, University of Salerno, Salerno, Italy; ^4^ Department of Molecular Cardiology, Heinrich Heine University, Düsseldorf, Germany; ^5^ Department of Experimental and Clinical Medicine, University of Catanzaro Magna Graecia, Catanzaro, Italy; ^6^ Department of Molecular Medicine and Medical Biotechnologies, School of Medicine and Surgery, University of Naples ‘Federico II’, Naples, Italy

**Keywords:** CD73, ecto-5′-nucleotidase, adenosine, sensitization, airways, inflammation, ovalbumin, allergy

## Abstract

The airways are a target tissue of type I allergies and atopy is the main etiological factor of bronchial asthma. A predisposition to allergy and individual response to allergens are dependent upon environmental and host factors. Early studies performed to clarify the role of extracellular adenosine in the airways highlighted the importance of adenosine-generating enzymes CD73, together with CD39, as an innate protection system against lung injury. In experimental animals, deletion of CD73 has been associated with immune and autoimmune diseases. Our experiments have been performed to investigate the role of CD73 in the assessment of allergic airway inflammation following sensitization. We found that in CD73^−/−^ mice sensitization, induced by subcutaneous ovalbumin (OVA) administration, increased signs of airway inflammation and atopy developed, characterized by high IgE plasma levels and increased pulmonary cytokines, reduced frequency of lung CD4^+^CD25+Foxp3+ T cells, but without bronchial hyperreactivity, compared to sensitized wild type mice. Our results provide evidence that the lack of CD73 causes an uncontrolled allergic sensitization, suggesting that CD73 is a key molecule at the interface between innate and adaptive immune response. The knowledge of host immune factors controlling allergic sensitization is of crucial importance and might help to find preventive interventions that could act before an allergy develops.

## Introduction

A predisposition for developing allergies is the result of a multifactorial interplay between genes and the environment. Intrinsic properties of exogenous proteins are important; however, host immune factors are crucial to explain the different response to allergens in individuals, some of whom do not develop allergies at all. Moreover, manifestations of allergies may be variable in different subjects, depending on individual, genetic, and environmental factors (van Ree et al., 2014).

There is much evidence that extracellular adenosine is an important regulator of the inflammatory/immune response (Antonioli et al., 2013). Extracellular adenosine derives from the hydrolysis of ATP mainly by the sequential action of CD39 and CD73 enzymes, the latter catalyzing the rate limiting step reaction (Zimmermann et al., 2012). CD73 is widely expressed on barrier cell types, such as endothelial and epithelial cells. It has been shown to have an important role in the control of vascular permeability following inflammation (Thompson et al., 2004).

Epithelial barrier damage may be associated with allergic sensitization and with the development of allergic manifestations (Fritz et al., 2008). It has been demonstrated that, in the murine model of contact hypersensitivity induced by trinitro-chloro benzene (TNCB), CD73 controls the sensitization against haptens by increasing adenosine accumulation in skin cells, such as keratinocytes and dendritic cells (DC). Interestingly, authors demonstrate that CD73 deficiency impairs the development of sensitization, rather than the effector phase induced by challenge (Neuberger et al., 2017).

Airways represent one of the tissues highly targeted by allergic sensitization, and bronchial asthma is a major clinical sign of type I allergies. The incidence of bronchial asthma development over other allergic manifestations in atopic patients is dependent upon environmental and genetic factors.

Recent findings indicate that, although atopy is the main risk factor, asthma cannot be considered a disease involving only the adaptive immune system, but instead that a crosstalk between innate and adaptive immune system is crucial to the assessment of allergic sensitization (Fritz et al., 2008; Holgate and Davies, 2009; Wenzel, 2012; Holgate et al., 2015). On these bases, it must be considered that stromal cells, epithelial cells, and immune-inflammatory cells all contribute to allergic sensitization.

Although much work has been published on the role of adenosine and its receptors in allergic asthma, and adenosine signaling might represent a therapeutic target (Brown et al., 2008; Polosa and Blackburn, 2009; Alfieri et al., 2012; Cicala and Ialenti, 2013), very little is known on the role of CD73 in the development of allergic sensitization as a major cause of those morphological and functional changes of airways that could facilitate asthma development (Schreiber et al., 2008; Neuberger et al., 2017).

The current study was designed to evaluate the role of CD73 in the development of allergic sensitization as a trigger of airway inflammation in mice; experiments were performed in a model of ovalbumin-sensitized wild type (WT) and CD73 deficient mice (CD73^−/−^). We demonstrate that allergic sensitization in CD73^−/−^ mice occurs and develops with increased airway inflammation and mucus production and increased plasma IgE levels, together with increased pulmonary cytokine levels, but without any increase in bronchial reactivity, compared to their WT counterparts.

Our results suggest that CD73 is essential to the assessment of a controlled sensitization; on the other hand, the loss of CD73 might prevent atopic subjects from bronchial hyperreactivity.

## Materials and Methods

### Animals

Female C57BL/6J (8 weeks old) were purchased from Charles River (Calco, Italy). Original CD73^−/−^ breeders were a kind gift from Professor Jurgen Schrader (Department of Molecular Cardiology, Heinrich Heine University, Düsseldorf, Germany). All experiments were approved by the Italian Ministry of Health and all methods were performed according to Italian (DL 26/2014) and European (n.63/2010/UE) guidelines and regulations.

### Allergic Sensitization

Mice were divided into sensitized and non-sensitized groups. Sensitization was induced by subcutaneous injection of 100 µg ovalbumin (OVA, grade V; Sigma Aldrich, Milan, Italy) emulsified with aluminum hydroxide (Al(OH)_3_, 13 mg/ml) on days 0 and 7. The non-sensitized groups (control) received an equal volume of aluminum hydroxide as previously described (Roviezzo et al., 2017). At days 14 and 21, mice were sacrificed, and main bronchi, pulmonary tissue, and blood were collected for functional and molecular studies.

### Western Blot

Pulmonary tissue from both control and OVA-sensitized WT mice was collected and transferred in FastPrep-24 lysing matrix tube (MP Biomedicals, Santa Ana, California, United States) together with ice-cold radioimmunoprecipitation assay buffer (RIPA buffer; Thermo Fisher Scientific, Monza, Italy) containing protease inhibitors cocktail (Sigma-Aldrich, Milan, Italy). Equal amounts of protein (50 µg) were separated on 8% SDS-PAGE gel and transferred to nitrocellulose membrane. Following blocking with 5% (w/v) non-fat dry milk in phosphate buffer saline (PBS) supplemented with 0.1% (v/v) tween 20, the nitrocellulose membrane was incubated overnight at 4°C with anti-mouse CD73 polyclonal goat antibody (1:200 dilution, Santa Cruz Biotechnology) and then it was incubated for 2 h at room temperature with the secondary antibody anti-goat IgG (1:2,000 dilution; Dako, CA, United States) conjugated with peroxidase. Successively, to confirm the equal protein loading, the membrane was stripped and incubated with anti β-actin monoclonal antibody (1:2,000 dilution, Santa Cruz Biotechnology) and subsequently with anti-mouse IgG conjugated to peroxidase (1:2,000 dilution, Dako, CA, United States) for 3 h at room temperature. Protein bands were detected using the ECL detection kit (Bio-Rad, Milan, Italy) and the ChemiDoc Imaging System (Bio-Rad, Italy). Densitometry was performed using Image Lab software (Bio-Rad, Milan, Italy).

### AMPase Activity

AMPase activity was assessed in lung homogenates and plasma from both control and sensitized WT mice, as a measure of CD73 activity, by colorimetric measurement of the inorganic phosphate (Pi) released following incubation with the substrate as previously described (Caiazzo et al., 2019). To have the net value of Pi produced following an enzymatic reaction, nonspecific Pi was released following incubation with the CD73 inhibitor, adenosine 5′- (α, β-methylene) diphosphate (APCP, 100 µM) in each sample was evaluated, and the value obtained was subtracted from the value obtained following incubation with AMP. Results were expressed as Pi released (pmol/min/µg protein).

### Enzyme-Linked Immunosorbent Assay

Total IgE levels were quantified in plasma samples while IL-4, IL-5, IL-10, and TGF-β1 cytokines were measured in lung homogenates using commercially available ELISA kits (BD Pharmingen, Franklin Lakes, NJ, United States or R&D Systems, Inc., Minneapolis, MN, United States) according to the manufacturer’s instructions.

### Flow Cytometry Analysis

Lungs from sensitized and control mice were digested with 0.5 U/ml collagenase (Sigma-Aldrich, Milan, Italy) and cell suspensions were passed through 70-µm cell strainers. T cells infiltrated into the lung were analyzed by flow cytometry using the antibodies CD3-PerCp (17A2), CD4-FITC (L3T4), CD8-PE (53–6.7), and CD25-PE (PC61), purchased from BD Biosciences; FoxP3- allophycocyanin (APC) (3G3) was purchased by Milteny Biotec. For lung Tregs detection, cells (5 × 10 ^5^) were incubated with anti-mouse CD3-PerCp (0.5 μg), anti-mouse CD4-FITC (0.5 μg), and anti-mouse CD25-PE (0.5 μg), followed by intracellular staining with anti-mouse FoxP3-APC antibody (0.5 μg) using FoxP3 staining buffer (eBioscience™ Intracellular Fixation & Permeabilization Buffer Set; Thermo Fisher Scientific, Monza, Italy). Cells were analyzed by flow cytometry using BD FACSAria flow cytometer and BD FACSDiva software (BD Biosciences), as previously described (Fiume et al., 2015).

### Lung Histology

Lung lobes were removed, fixed in 4% formalin, and embedded in paraffin. Tissue was then sectioned (7 μm thickness) and stained with haematoxylin and eosin (H & E) for morphological analysis. Additional sections were stained with Alcian Blue/Periodic Acid-Schiff (AB/PAS) for goblet cell evaluation. The severity of lung inflammation was visually scored in a blinded fashion on H & E - stained slides and graded through a subjective, semiquantitative five-point scale as previously described (Lafkas et al., 2015): 0, normal lung (no inflammatory infiltrates); 1, minimal disease (infrequent sparsely scattered inflammatory cells); 2, mild (light perivascular or peribronchiolar involvement); 3, moderate (many vessels and airways affected by substantial numbers of inflammatory cells); and 4, severe (generalized accumulations of perivascular or peribronchiolar inflammatory cells with frequent circumferential or bridging infiltrates, or both). Severity of bronchiolar epithelial goblet cell hyperplasia was visually scored in a blinded fashion on AB/PAS-stained slides through a subjective, semiquantitative four-point scale, according to Sun and co-workers(2015), as follows: 0, negative (rare or no AB/PAS-positive cells); 1, low (scattered AB/PAS-positive cells goblet constituting <25% of the epithelium); 2, moderate (more frequent AB/PAS-positive cells constituting 25–50% of the epithelium); and 3, high (many AB/PAS-positive cells constituting >50% of the epithelium) (Sun et al., 2015).

### Airway Responsiveness Measurements

Different groups of animals were sensitized as described above. At 21 days, main bronchi collected from both sensitized and control mice were rapidly dissected and cleaned from fat and connective tissue. Rings of 1–2 mm length were cut and mounted in 2.5 ml isolated organ baths containing Krebs solution, at 37°C, oxygenated (95% O_2_ and 5% CO_2_), and connected to an isometric force transducer (type 7006, Ugo Basile, Comerio, Italy) associated to a Powerlab 800 (AD Instruments). Bronchial reactivity to cumulative concentrations of carbachol (10^−9^ – 3 × 10^−6^ M) was evaluated. Results were expressed as dyne per mg of tissue (Morello et al., 2005).

### Statistical Analysis

All results are presented as means ± standard error (SE). Statistical analysis was performed through Student’s *t*-test for unpaired data or by one- or two-way ANOVA for multiple comparisons followed by Bonferroni post-hoc test. A *p* value <0.05 was considered statistically significant.

## Results

### Ovalbumin Sensitization Increases CD73 Expression and Activity

**FIGURE 1:**
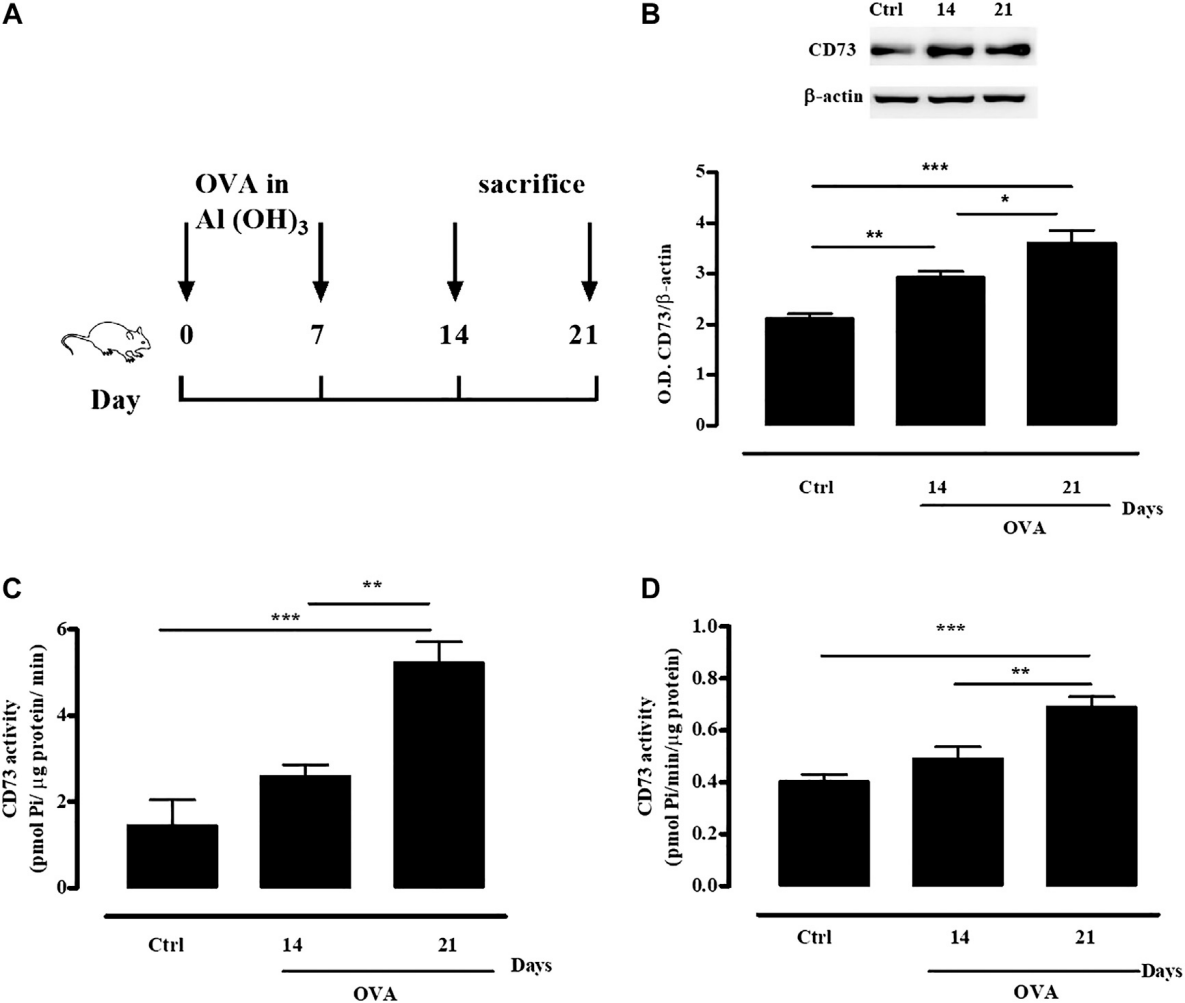
OVA sensitization increases CD73 expression and activity. **(A)** Experimental protocol for OVA-induced sensitization in WT and CD73^−/−^ mice. Mice were sensitized or not with OVA as described in *Materials and Methods*. **(B)** CD73 expression in whole lung tissue from both control and OVA-sensitized WT mice was quantified by Western blots. Means ± SE of eight mice per group. *p < 0.05, **p < 0.01 and ***p < 0.001 (one-way ANOVA followed by Bonferroni’s Multiple Comparison Test). CD73 specific activity was measured in lung homogenates **(C)** and plasma **(D)** from both control and OVA-sensitized WT mice by Malachite green assay. All results are expressed as mean ± SE of six mice per group. **p < 0.01 and ***p < 0.001 (one-way ANOVA followed by Bonferroni’s Multiple Comparison Test).

Following mice sensitization with OVA, administered subcutaneously twice (day 0 and 7, Figure 1A), the expression of CD73 was increased in whole lung tissue both at 14 and 21 days, compared to the expression of control tissue from non-sensitized mice (Figure 1B); in accordance, pulmonary and plasmatic AMPase activity was significantly increased following OVA sensitization (Figures 1C,D, respectively). Results obtained by sample incubation with APCP demonstrated that it was dependent upon CD73 (data not shown).

### Increased IgE Levels in Ovalbumin-Sensitized CD73^−/−^ Mice

Sensitization with OVA increased plasma IgE levels at 14 days, as already described (Rossi et al., 2017).

**FIGURE 2:**
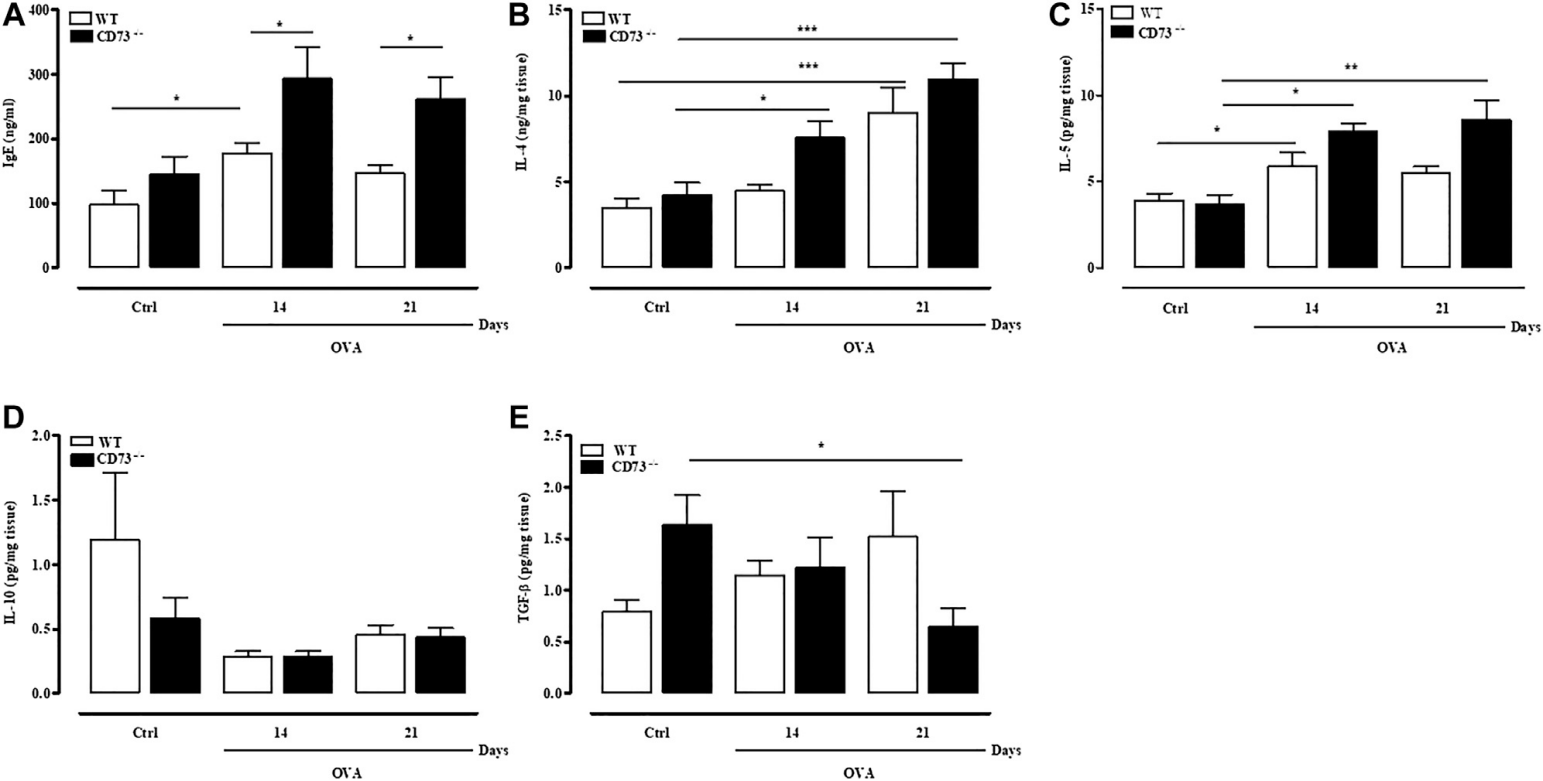
CD73 deficiency further increases IgE, IL-4, and IL-5 levels induced by OVA sensitization and decreases TGF-β pulmonary levels after OVA-sensitization. Plasma levels of total IgE **(A)** and pulmonary levels of IL-4 **(B)**, IL-5 **(C)**, IL-10 **(D)**, and TGF-β **(E)** were measured in both control and OVA-sensitized WT and CD73^−/−^ mice by ELISA. All results are expressed as mean ± SE of n = 6–12 mice per group. *p < 0.05, **p < 0.01 and ***p < 0.001 (Student’s t-test for unpaired data and one-way ANOVA followed by Bonferroni’s Multiple Comparison Test).

In CD73^−/−^ mice there was a significant increase of plasma IgE levels, both at 14- and 21-days following sensitization, compared to IgE plasma levels detected in WT mice (Figure 2A).

### Increased Levels of IL-4 and IL-5 and Reduced TGF-β in Lungs of Ovalbumin-Sensitized CD73^−/−^ Mice

Pulmonary cytokine levels were evaluated in WT and CD73^−/−^ mice following sensitization. In WT mice pulmonary levels of IL-4 peaked 21 days following sensitization; in CD73^−/−^ mice, IL-4 levels increased starting from 14 days following sensitization (Figure 2B). Similarly, IL-5 pulmonary levels were significantly increased in CD73^−/−^ compared to control value at 14- and 21-days following sensitization (Figure 2C). There was no difference in IL-10 pulmonary levels between sensitized WT and CD73^−/−^ mice (Figure 2D). Levels of TGF-β tended to increase in sensitized WT mice compared to control value, while in CD73^−/−^ mice they were progressively reduced (Figure 2E).

### Increased Pulmonary Inflammation and Mucus Production in Ovalbumin-Sensitized CD73^−/−^ Mice

**FIGURE 3:**
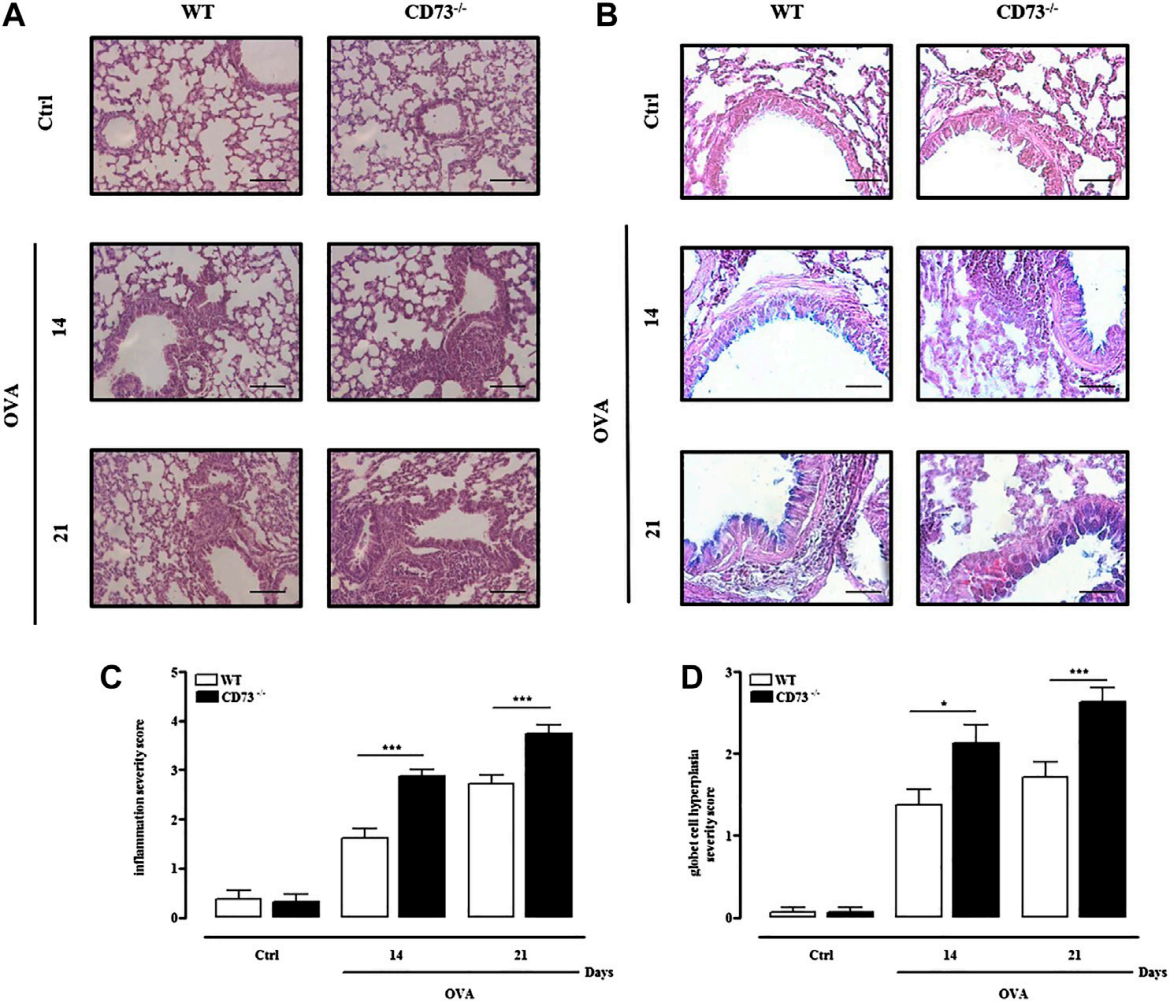
CD73 deficiency further increases lung inflammation and goblet cell hyperplasia induced by OVA. Histological examination of lung tissues from both control and OVA-sensitized WT and CD73^−/−^ mice was performed via staining with **(A)** hematoxylin and eosin (H & E), and **(B)** Alcian Blue/Periodic acid-Schiff (AB/PAS) staining. Scale bar, 100 μm **(A)** and 50 μm **(B)**. **(C)** Lung inflammation scores. **(D)** Scores of airway epithelium graded for goblet cell hyperplasia. All results are expressed as mean ± SE of n = 8 mice per group. *p < 0.05 and ***p < 0.001 (one-way ANOVA followed by Bonferroni’s Multiple Comparison Test).

Airway inflammation was assessed in H & E stained tissue sections from lungs of WT and CD73^−/−^ mice. Morphological analysis of lungs evidenced a massive cell infiltration in lungs from CD73^−/−^ mice following sensitization compared to WT mice (Figure 3A), as evidenced by the mean inflammation score (Figure 3C). AB/PAS staining evidenced goblet cell hyperplasia following sensitization, which was particularly evident in CD73^−/−^ mice (Figures 3B,D).

### Pulmonary CD4^+^CD25^+^Foxp3^+^Treg Frequency Is Reduced in CD73^−/−^ Mice Following Ovalbumin-Sensitization

**FIGURE 4:**
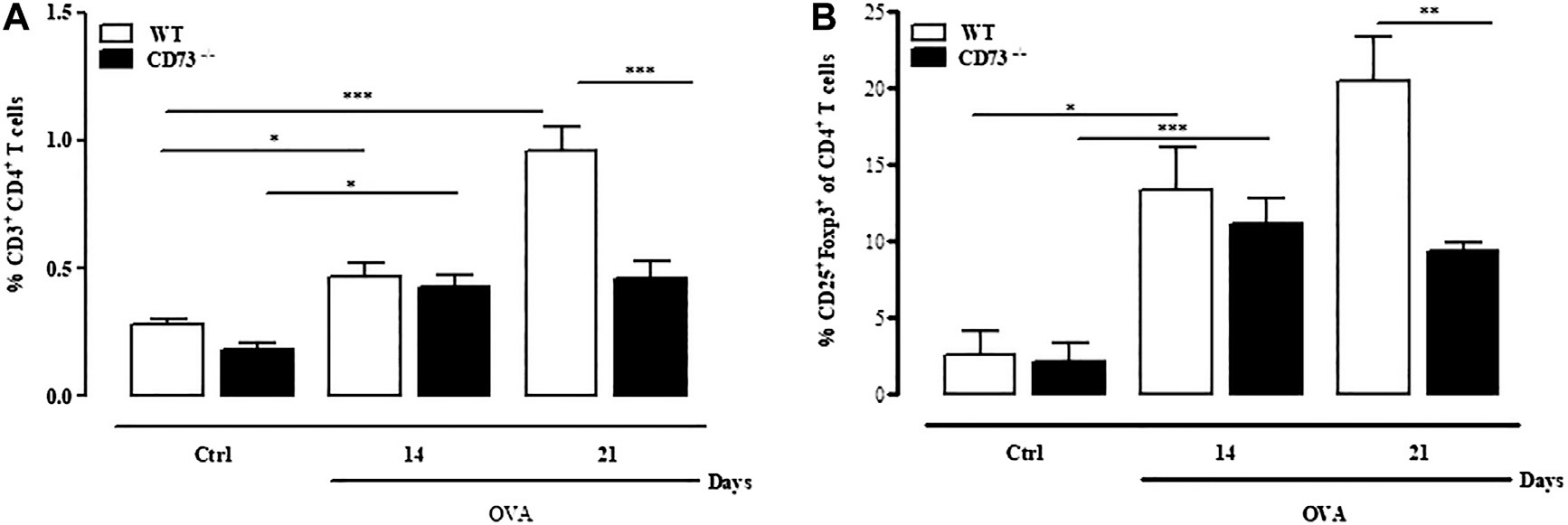
CD73 deficiency reduces the frequency of CD4^+^CD25+Foxp3+ regulatory T cells (Tregs) infiltrating the lung. Lungs from both control and OVA-sensitized WT and CD73−/− were isolated and analyzed using FACS to determine the phenotype of Tregs cells. Percentage of CD3+CD4+ T cells **(A)** and CD4+CD25+Foxp3+ Tregs **(B)** in the lungs. All results are expressed as mean ± SE of n = 6 mice per group. *p < 0.05, **p < 0.01 and ***p < 0.001 (Student’s t-test for unpaired data and Bonferroni’s Multiple Comparison Test).

Evidence that sensitization caused an exacerbated pulmonary inflammation in CD73^−/−^ mice compared to WT mice suggests that in the absence of CD73 there is a disregulation of the immune response. Analysis of total lung-infiltrating T cells (Figure 4A) reveals that the percentage of CD3^+^CD4^+^T cells significantly increased in WT mice after OVA sensitization compared to control, reaching the highest level at day 21 Conversely, in CD73^−/−^ mice the frequency of total CD3^+^CD4^+^T cells slightly increased at day 14 and day 21 after OVA sensitization compared to their counterparts (Figure 4A); however, compared to WT mice, the frequency of these cells was significantly reduced (Figure 4A). Among CD3^+^CD4^+^T cells the frequency of CD4^+^CD25^+^Foxp3^+^ T reg cells subpopulation increased in WT mice following sensitization compared to control (Figure 4B) and remained higher still than those observed in CD73^−/−^ mice (Figure 4B). There was no difference in the percentage of total CD3^+^CD4^+^T cells nor Tregs between control WT and CD73^−/−^ mice (Figures 4A,B).

### Absence of Bronchial Hyperreactivity to Carbachol of Sensitized CD 73^−/−^ Mice

**FIGURE 5:**
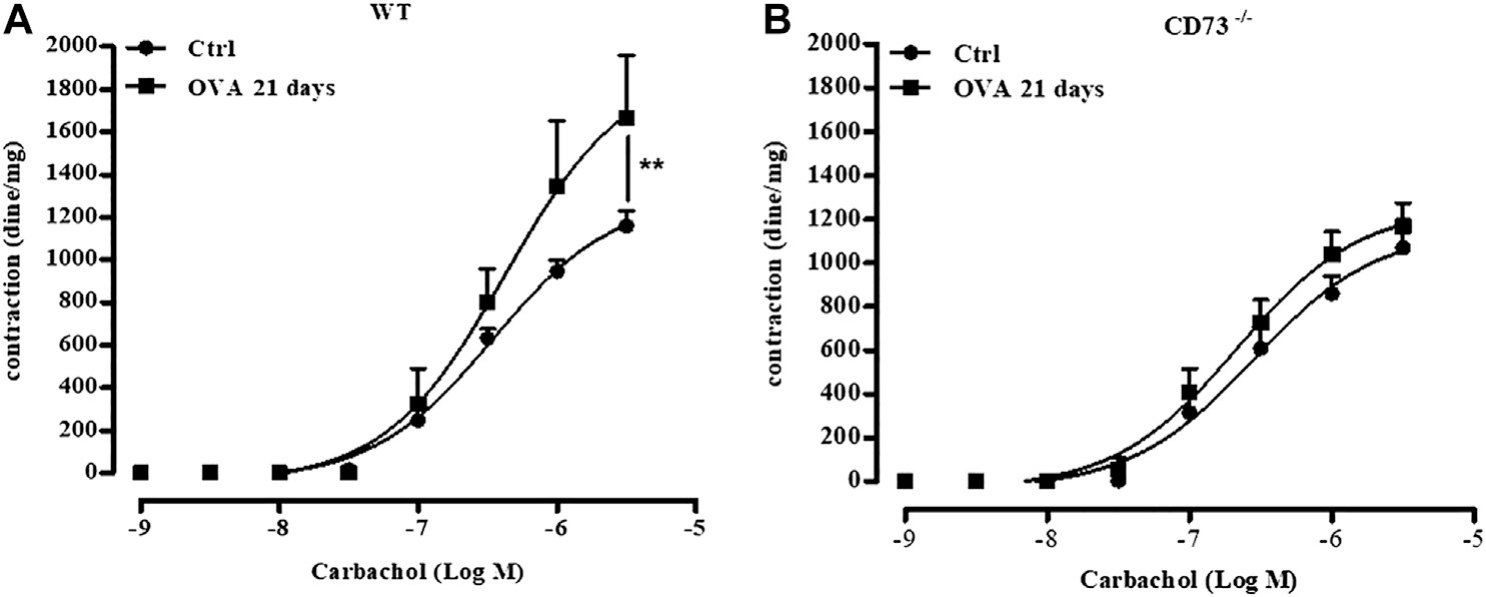
CD73 deficiency does not increase bronchial reactivity to carbachol induced by OVA-sensitization. Carbachol-induced contractions of bronchi collected from both control and from 21 days OVA-sensitized WT **(A)** and CD73−/− **(B)** mice. The concentration-response curves for carbachol **(A,B)** have been obtained by a non-linear regression analysis. All results are expressed as mean ± SE of n = 6–12 per group. **p < 0.01 (Two-way ANOVA followed by Bonferroni).

We finally evaluated the reactivity to carbachol of bronchi isolated 21 days following sensitization from WT and CD73^−/−^ mice. Sensitization increased reactivity to carbachol of bronchi from WT mice, while it did not affect bronchial reactivity to carbachol in CD73^−/−^ mice (Figures 5A,B).

## Discussion

The aim of the present work was to investigate the role played by CD73, the key enzyme in the extracellular adenosine accumulation, in the development of features of allergic sensitization, a risk factor for asthma and allergic diseases.

We found that in OVA-sensitized C57Bl/6J mice, susceptible to airway inflammation and hyperreactivity, as previously described (Rossi et al., 2017), pulmonary expression of CD73 was significantly up-regulated; concomitantly, there was increased pulmonary and plasma CD73 activity compared to control, non-sensitized mice. This finding is consistent with data demonstrating that CD73 upregulation following injury may represent a tissue self-protective strategy, providing an increase of extracellular adenosine levels that, in turn, may control inflammation and tissue damage; hence, inhibition of CD73 activity may exacerbate ongoing inflammation (Caiazzo et al., 2019). On the other hand, there is evidence that the increased CD73 expression and activity in inflamed tissue may cause persistent extracellular adenosine accumulation that, in turn, may be detrimental, driving the tissue toward chronic inflammation and fibrosis (Cronstein and Sitkovsky, 2017; Le et al., 2019). Thus, under some aspects, CD73/adenosine appears to be a double-edged sword: dissecting the role of this pathway in the assessment of the host response to an inflammatory trigger stimulus from its role on the ongoing tissue inflammation is crucial to find preventive and therapeutic strategies.

We found that OVA-sensitized CD73^−/−^ mice produced increased levels of Th2 cytokines, IL-4, and IL-5 in the lung compared to Th2 cytokines produced by sensitized WT mice. Pulmonary IL-13 levels were undetectable in all groups of animals(data not shown). IL-4 is a cytokine that plays a central role in allergic airways, it may also derive from mast cells and basophils and it is crucial for Th2 differentiation from naïve Th cells (KleinJan 2016). Furthermore, IL-4 is considered to play a central role in the perpetuation of IgE - mediated allergic diseases (Herrick and Bottomly, 2003; Caminati et al., 2018). In our experiments, sensitized CD73^−/−^ mice showed features of increased atopy, characterized by increased pulmonary IL-4 levels together with persistent high IgE plasma levels, compared to their WT counterparts.

IL-5 is a cytokine derived from a wide variety of stromal and immune/inflammatory cells, beside Th2 cells, known to be mainly involved in eosinophil proliferation, differentiation, and recruitment in allergic diseases and asthma. There is also evidence that IL-5 derived by airway epithelial cells greatly contributes to local immune response and pathological changes in allergic airway disease in mice (Lee et al., 1997; Wu et al., 2010). In sensitized CD73^−/−^ mice, associated with increased airway inflammation and mucus production and goblet cell hyperplasia, we found elevated pulmonary IL-5 levels that are likely produced from stromal cells, or other cell subsets, such as innate lymphoid cells, lacking the inhibitory control of CD73-derived adenosine (Csoka et al., 2008; Csoka et al., 2018). A worsening of pulmonary inflammation in the absence of CD73 has already been demonstrated in other animal models, where different damaging agents have been used (Volmer et al., 2006; Eckle et al., 2007; Ehrentraut et al., 2013; Li et al., 2017).

Sensitization increased the frequency of CD4^+^CD25^+^Foxp3^+^ T cells in the lungs of WT mice, but not in the lungs of CD73^−/−^ mice, in concomitance with a slight increase of TGF-β levels. There is much evidence that regulatory T cells are important modulators of immune response and allergic inflammation. The surrounding microenvironment plays a major role in directing T cells toward a specific subset; such evidence is particularly pertinent to airways, since they represent a specialized site in continuous connection with an external atmosphere (Ray et al., 2010; Noval Rivas and Chatila, 2016). Our findings obtained in CD73^−/−^ mice may be consistent with the lack of local adenosine known to drive T cell polarization and Treg expansion, during sensitization, directly acting on T cells or, indirectly, influencing cytokine production by macrophages or other antigen presenting cells (APC) (Ohta et al., 2012; Pei and Linden, 2016). There is also evidence that CD73 is highly expressed on Foxp3+ Tregs and its enzymatic activity together with CD39 enzymatic activity is critical for Treg – mediated immunosuppression (Ehrentraut et al., 2013; Ohta and Sitkovsky, 2014). Consistently, it has been demonstrated that the immunoregulatory role of vitamin D3 might be dependent upon its ability to up-regulate CD73 on human Treg via TGF-β (Mann et al., 2015).

To investigate whether biochemical and morphological differences observed between sensitized CD73^−/−^ and WT mice reflected in airway functional difference, we evaluated the bronchial response to carbachol, *in vitro*, since, in this model of OVA sensitized mice, it has been demonstrated that bronchial hyperreactivity occurs 21 days following sensitization (Roviezzo et al., 2015; Rossi et al., 2017). It is worth noting that the increased lung inflammation observed in sensitized mice lacking CD73 is not reflected in airway hyperreactivity. In humans, it has been demonstrated that the degree of bronchial hyperreactivity is associated with the degree of atopy (Grootendorst and Rabe, 2004). Here, we show that in absence of CD73, despite signs of increased atopy, characterized by increased airway inflammation and IgE levels, bronchial hyperreactivity following sensitization is absent.

On the basis of our results, we could speculate that during the sensitization period, the lack of CD73 would be detrimental, likely because the reduced physiological adenosine availability causes the lack of an “immunosurveillance” mechanism; conversely, into inflamed allergic airways, the lack of CD73 could prevent bronchial hyperreactivity by preventing high adenosine pulmonary accumulation. It must also be considered that CD73 is the ectoenzyme that ultimately controls the ratio of extracellular ATP vs. adenosine; thus, in the absence of CD73 the airway microenvironment would be transformed, since the lack of AMP hydrolysis may result in altered levels of upstream nucleotides, ADP and ATP. On this basis, the loss of adenosine-mediated effects would give the way to ATP - and ADP - mediated inflammatory effects (Thompson et al., 2004; Koszalka et al., 2004; Colgan et al., 2006). Recently, it has been demonstrated that ATP through P2Y1 receptor activation on airway vagal sensory neurons activates protective reflexes of the upper airways, like reflexes to avoid pulmonary aspiration, such as pharyngeal swallowing. A hyperstimulation of this neuronal circuits may contribute to airway diseases, such as asthma and COPD (Prescott et al., 2020). Further work will be necessary for a complete investigation of the control of the ATP/ADP/adenosine balance in airways.

In conclusion, our results support a model in which CD73^−/−^ mice respond to the assessment of allergic sensitization with a more robust inflammation in airways, representing a target tissue of sensitization and type I allergy, with increased plasma IgE levels and reduced pulmonary Treg expansion. Recently a great effort has been made not to confine allergic airway inflammation into the paradigm of Th2 inflammation, but to have a wide view of the phenomenon that subtends different asthma phenotypes (Wenzel, 2012; Hirose et al., 2017). In this respect, we could speculate that CD73, an important molecule present on epithelial barriers and immune-inflammatory cells, plays a crucial role at the interface between innate and adaptive response in the assessment of sensitization and allergic airway inflammation. We could look at CD73 expression on immune cells or at its soluble form as a biomarker prognostic for allergy and atopy; in addition, increasing the function of this pathway might stimulate individual immune responses in healthy subjects. Our results may have implications for preventive and therapeutic strategies in allergic airway inflammation.

## Data Availability Statement

The raw data supporting the conclusions of this article will be made available by the authors, without undue reservation, to any qualified researcher.

## Author Contributions

EC, IC, MR, RT, GF, and CC (affiliation 6) performed experiments; EC, SM, and CC (affiliation 1) conceived the study and analyzed results; EC and CC (affiliation 1) wrote the manuscript; AI, JS, GF, FR, and SM contributed to figure revision and data presentation; all authors reviewed the manuscript.

## Funding

This work was supported by the Grant of University of Naples Federico II (Research Program 2017 – 2019; DR 409 del 7 Febbraio 2017).

## Conflict of Interest

The authors declare that the research was conducted in the absence of any commercial or financial relationships that could be construed as a potential conflict of interest.

## References

[B1] Alfieri A. Parisi A. Maione F. Grassia G. Morello S. Ialenti A. Mascolo N. Cicala C. (2012). Hyperresponsiveness to adenosine in sensitized Wistar rats over-expressing A1 receptor. Eur J Pharmacol. 695, 120 - 125. 10.1016/j.ejphar.2012.09.002. 23000391

[B2] Antonioli L. Blandizzi C. Pacher P. Haskó G. (2013). Immunity, inflammation and cancer: a leading role for adenosine. Nat Rev Cancer. 13, 842-857. 10.1038/nrc3613. 24226193

[B3] Brown R.A. Spina D. Page C.P. (2008). Adenosine receptors and asthma. Br J Pharmacol. 153, (Suppl 1) S446-S456. 10.1038/bjp.2008.22. 18311158 PMC2268070

[B4] Caiazzo E. Morello S. Carnuccio R. Ialenti A. Cicala C. (2019). The Ecto-5'-Nucleotidase/CD73 Inhibitor, α,β-Methylene Adenosine 5'-Diphosphate, Exacerbates Carrageenan-Induced Pleurisy in Rat. Front Pharmacol. 10:775. 10.3389/fphar.2019.00775. 31354490 PMC6637294

[B5] Caminati M. Pham D.L. Bagnasco D. Canonica G.W. (2018). Type 2 immunity in asthma. World Allergy Organ J. 11:13 10.1186/s40413-018-0192-5. 29988331 PMC6020328

[B6] Cicala C. Ialenti A. (2013). Adenosine signaling in airways: toward a promising antiasthmatic approach. Eur J Pharmacol. 714, 522 - 525. 10.1016/j.ejphar.2013.06.033. 23850943

[B7] Colgan S.P. Eltzschig H.K. Eckle T. Thompson L.F. (2006). Physiological roles for ecto-5'-nucleotidase (CD73). Purinergic Signal. 2(2), 351-360. 10.1007/s11302-005-5302-5. 18404475 PMC2254482

[B8] Cronstein B.N,. Sitkovsky M. (2017). Adenosine and adenosine receptors in the pathogenesis and treatment of rheumatic diseases. Nat Rev Rheumatol. 13, 41–51. 10.1038/nrrheum.2016.178 27829671 PMC5173391

[B9] Csóka B. Himer L. Selmeczy Z. Vizi E.S. Pacher P. Ledent C. (2008). Adenosine A2A receptor activation inhibits T helper 1 and T helper 2 cell development and effector function. FASEB J. 22, 3491–3499. 10.1096/fj.08-107458. 18625677 PMC2537430

[B10] Csóka B. Németh Z.H. Duerr C.U. Fritz J.H. Pacher P. Haskó G. (2018). Adenosine receptors differentially regulate type 2 cytokine production by IL-33-activated bone marrow cells, ILC2s, and macrophages. FASEB J. 32, 829–837. 10.1096/fj.201700770R. 28982732 PMC5888397

[B11] Eckle T. Füllbier L. Wehrmann M. Khoury J. Mittelbronn M. Ibla J. (2007). Identification of ectonucleotidases CD39 and CD73 in innate protection during acute lung injury. J Immunol. 178, 8127 - 8137. 10.4049/jimmunol.178.12.8127. 17548651

[B12] Ehrentraut H. Clambey E.T. McNamee E.N. Brodsky K.S. Ehrentraut S.F. Poth J.M. (2013). CD73+ regulatory T cells contribute to adenosine-mediated resolution of acute lung injury. FASEB J. 27, 2207 - 2219. 10.1096/fj.12-225201. 23413361 PMC3659359

[B13] Fiume G. Scialdone A. Albano F. Rossi A. Tuccillo F.M. Rea D. (2015). Impairment of T cell development and acute inflammatory response in HIV-1 Tat transgenic mice. Sci.Rep. 5, 138642015. 10.1038/srep13864. PMC4561375 26343909

[B14] Fritz J.H. Le Bourhis L. Magalhaes J.G. Philpott D.J. (2008). Innate immune recognition at the epithelial barrier drives adaptive immunity: APCs take the back seat. Trends Immunol. 29, 41-49. 10.1016/j.it.2007.10.002. 18054284

[B15] Grootendorst D.C. Rabe K.F. (2004). Mechanisms of bronchial hyperreactivity in asthma and chronic obstructive pulmonary disease. Proc Am Thorac Soc. 1, 77 - 87. 10.1513/pats.2306025. 16113417

[B16] Herrick C.A. Bottomly K. (2003). To respond or not to respond: T cells in allergic asthma. Nat Rev Immunol. 3, 405 ‐ 412. 10.1038/nri1084. 12766762

[B17] Hirose K. Iwata A. Tamachi T. Nakajima H. (2017). Allergic airway inflammation: key players beyond the Th2 cell pathway. Immunol Rev. 278, 145 - 161. 10.1111/imr.12540. 28658544

[B18] Holgate S.T. Davies D.E. Rethinking the pathogenesis of asthma. (2009). Immunity. 31, 362-367. 10.1016/j.immuni.2009.08.013. 19766079

[B19] Holgate S.T. Wenzel S. Postma D.S. Weiss S.T. Renz H. Sly P.D. (2015). Asthma. Nat Rev Dis Primers. 1:15025. 10.1038/nrdp.2015.25. 27189668 PMC7096989

[B20] KleinJan A. (2016). Airway inflammation in asthma: key players beyond the Th2 pathway. Curr Opin Pulm Med. 22, 46 – 52. 10.1097/MCP.0000000000000224. 26574718

[B21] Koszalka P. Ozuyaman B. Huo Y. Zernecke A. Flogel U. Braun N. Buchheiser A. Decking U.K. Smith M.L. Sevigny J. Gear A. (2004). Targeted disruption of CD73/ecto-5′-nucleotidase alters thromboregulation and augments vascular inflammatory response. Circ Res 95,814–821. 15358667 10.1161/01.RES.0000144796.82787.6f

[B22] Lafkas D. Shelton A. Chiu C. de Leon Boenig G. Chen Y. Stawicki S.S. (2015). Therapeutic antibodies reveal Notch control of transdifferentiation in the adult lung. Nature. 528, 127-131. 10.1038/nature15715. 26580007

[B23] Le T.T. Berg N.K. Harting M.T. Li X. Eltzschig H.K. Yuan X. (2019). Purinergic Signaling in Pulmonary Inflammation. Front Immunol. 10:1633. 10.3389/fimmu.2019.01633. 31379836 PMC6646739

[B24] Lee J.J. McGarry M.P. Farmer S.C. Denzler K.L. Larson K.A. Carrigan P.E. (1997). Interleukin-5 expression in the lung epithelium of transgenic mice leads to pulmonary changes pathognomonic of asthma. J Exp Med. 185, 2143 ‐ 2156. 10.1084/jem.185.12.2143. 9182686 PMC2196351

[B25] Li H. Karmouty-Quintana H. Chen N.Y. Mills T. Molina J. Blackburn M.R. Davies J. (2017). Loss of CD73-mediated extracellular adenosine production exacerbates inflammation and abnormal alveolar development in newborn mice exposed to prolonged hyperoxia. Pediatr Res. 10.1038/pr.2017.176. 28832580

[B26] Mann E.H. Chambers E.S. Chen Y.H. Richards D.F. Hawrylowicz C.M. (2015). 1α,25-dihydroxyvitamin D3 acts via transforming growth factor-β to up-regulate expression of immunosuppressive CD73 on human CD4+ Foxp3- T cells. Immunology. 146, 423 - 431. 10.1111/imm.12519. 26251265 PMC4610631

[B27] Morello S. Vellecco V. Roviezzo F. Maffia P. Cuzzocrea S. Cirino G. Cicala C. (2005). A protective role for proteinase activated receptor 2 in airways of lipopolysaccaride-treated rats. Biochem. Pharmacol. 71, 223 - 230. 10.1016/j.bcp.2005.10.016. 16300746

[B28] Neuberger A. Ring S. Silva-Vilches C. Schrader J. Enk A. Mahnke K. (2017). Expression of CD73 slows down migration of skin dendritic cells, affecting the sensitization phase of contact hypersensitivity reactions in mice. J Dermatol Sci. 87, 292-299 (2017). 10.1016/j.jdermsci.2017.07.002. 28743609

[B29] Noval Rivas M. Chatila T.A.(2016). Regulatory T cells in allergic diseases. J Allergy Clin Immunol. 138, 639 - 652. 10.1016/j.jaci.2016.06.003. 27596705 PMC5023156

[B30] Ohta A. Kini R. Ohta A. Subramanian M. Madasu M. Sitkovsky M. (2012). The development and immunosuppressive functions of CD4(+) CD25(+) FoxP3(+) regulatory T cells are under influence of the adenosine-A2A adenosine receptor pathway. Front Immunol. 3:190. 10.3389/fimmu.2012.00190. 22783261 PMC3389649

[B31] Ohta A. Sitkovsky M. (2014). Extracellular adenosine-mediated modulation of regulatory T cells. Front Immunol. 5:304 10.3389/fimmu.2014.00304. 25071765 PMC4091046

[B32] Pei H. Linden J. (2016). Adenosine influences myeloid cells to inhibit aeroallergen sensitization. Am J Physiol Lung Cell Mol Physiol. 310, L985 - L992. 10.1152/ajplung.00330.2015. 27016586 PMC4896100

[B33] Polosa R. Blackburn M.R. (2009). Adenosine receptors as targets for therapeutic intervention in asthma and chronic obstructive pulmonary disease. Trends Pharmacol Sci. 30, 528 - 535. 10.1016/j.tips.2009.07.005. 19762093 PMC2778268

[B34] Prescott S.L. Umans B.D. Williams E.K. Brust R.D. Liberles S.D. (2020). An Airway Protection Program Revealed by Sweeping Genetic Control of Vagal Afferents. Cell 181(3), 574-589.e514. 10.1016/j.cell.2020.03.004. 32259485 PMC7197391

[B35] Ray A. Khare A. Krishnamoorthy N. Qi Z. Ray P. (2010). Regulatory T cells in many flavors control asthma. Mucosal Immunol. 3, 216 ‐ 229. 10.1038/mi.2010.4. 20164832 PMC3039023

[B36] Rossi A. Caiazzo E. Bilancia R. Riemma M.A. Pagano E. Cicala C. (2017). Salvinorin A Inhibits Airway Hyperreactivity Induced by Ovalbumin Sensitization. Front Pharmacol. 7: 525 10.3389/fphar.2016.00525. 28133450 PMC5233683

[B37] Roviezzo F. Bertolino A. Sorrentino R. Terlizzi M. Matteis M. Calderone V. (2015). Hydrogen sulfide inhalation ameliorates allergen induced airway hypereactivity by modulating mast cell activation. Pharmacol Res. 100, 85 - 92. 10.1016/j.phrs.2015.07.032 26241177

[B38] Roviezzo F. Rossi A. Caiazzo E. Orlando P. Riemma M.A. Iacono V.M. (2017). Palmitoylethanolamide Supplementation during Sensitization Prevents Airway Allergic Symptoms in the Mouse. Front Pharmacol. 8, 857. 10.3389/fphar.2017.00857. 29311913 PMC5732963

[B39] Schreiber R. Castrop H. Kunzelmann K. (2008). Allergen-induced airway hyperresponsiveness is absent in ecto-5'-nucleotidase (CD73)-deficient mice. Pflugers Arch. 457, 431‐440. 10.1007/s00424-008-0543-0. 18607626

[B40] Sun Y. Peng I. Webster J.D. Suto E. Lesch J. (2015). Inhibition of the kinase ITK in a mouse model of asthma reduces cell death and fails to inhibit the inflammatory response. Sci Signal. 8:ra122. 10.1126/scisignal.aab0949. 26628680

[B41] Thompson L.F. Eltzschig H.K. Ibla J.C. Van De Wiele C.J. Resta R. Morote-Garcia J.C. Colgan S.P. (2004). Crucial role for ecto-5'-nucleotidase (CD73) in vascular leakage during hypoxia. J. Exp. Med. 200, 1395-1405. 10.1084/jem.20040915. 15583013 PMC1237012

[B42] van Ree R. Hummelshøj L. Plantinga M. Poulsen L.K. Swindle E. (2014) . Allergic sensitization: host-immune factors. Clin Transl Allergy. 4, 12 10.1186/2045-7022-4-12. 24735802 PMC3989850

[B43] Volmer J.B. Thompson LF. Blackburn M.R. (2006). Ecto-5'-nucleotidase (CD73)-mediated adenosine production is tissue protective in a model of bleomycin-induced lung injury. J Immunol. 176, 4449 - 4458. 10.4049/jimmunol.176.7.4449. 16547283

[B44] Wenzel S.E. (2012). Asthma phenotypes: the evolution from clinical to molecular approaches. Nat Med. 18, 716 - 725. 10.1038/nm.2678. 22561835

[B45] Wu C.A. Peluso J.J. Zhu L. Lingenheld E.G. Walker S.T. Puddington L. (2010). Bronchial epithelial cells produce IL-5: implications for local immune responses in the airways. Cell Immunol. 264, 32-41. 10.1016/j.cellimm.2010.04.008. 20494340 PMC2902557

[B46] Zimmermann H. Zebisch M. Sträter N. (2012). Cellular function and molecular structure of ecto-nucleotidases. Purinergic Signal. 8, 437-502. 10.1007/s11302-012-9309-4. 22555564 PMC3360096

